# DFT study of binding and electron transfer from colorless aromatic pollutants to a TiO_2_ nanocluster: Application to photocatalytic degradation under visible light irradiation

**DOI:** 10.3762/bjnano.5.115

**Published:** 2014-07-11

**Authors:** Corneliu I Oprea, Petre Panait, Mihai A Gîrţu

**Affiliations:** 1 Department of Physics, Ovidius University of Constanţa, Constanţa 900527, Romania

**Keywords:** colorless aromatic pollutants, density functional theory, photocatalytic degradation, titanium dioxide, visible light irradiation

## Abstract

We report results of density functional theory (DFT) calculations on some colorless aromatic systems adsorbed on a TiO_2_ nanocluster, in order to explain experimental results regarding the photocatalytic degradation of these pollutants under visible light irradiation. Based on our modeling, we are able to clarify why transparent pollutants can degrade under visible light in the presence of a catalyst that absorbs only in the UV, to explain experimental data regarding differences in the efficiency of the degradation process, and to state the key requirements for effective water-cleaning. For that purpose, we analyze the absorption spectrum of the free and adsorbed molecules, the binding configurations, the matching of the energy levels with the oxide catalyst and the likelihood of the charge-transfer to the substrate. The comparison between several colorless aniline and phenolic systems allows a correlation between the chemical structure and the degradation rate of these pollutants.

## Introduction

Titania, TiO_2_, has been widely used as photocatalyst for environmental applications [1–6], particularly for waste water purification. Due to its large band gap TiO_2_ absorbs only UV radiation, a fact that limits the efficiency and keeps the costs of the photocatalytic degradation of environmental pollutants high. To be used under visible light irradiation, in the range of wavelengths where the solar spectrum has its maximum, the electronic band structure of the photocatalyst has to be modified in various ways [6]. Alternative approaches to the modification of the TiO_2_ photocatalyst are the self-sensitized degradation of dyes which absorb visible light [7–8] and the photocatalytic degradation of colorless organic compounds by formation of a charge-transfer-complex, CTC [9–10]. The assumption of a surface CTC in the visible light catalysis was supported by subsequent work on various other types of systems, such as phenolic compounds [11–12], fluoroquinolone antibacterial agents [13], and various colorless aromatic pollutants [14].

Despite the extensive experimental work, the role of the key factors that influence the microscopic mechanism of the photocatalytic processes is not entirely understood. Our goal is to answer a few key questions, regarding photocatalytic degradation: i) Why can transparent pollutants degrade under visible light in the presence of a catalyst that absorbs only in the UV? ii) Why are some pollutants degraded more efficiently than others? iii) What are the requirements for an effective water-cleaning process? To answer these questions we start from an analogy with the photoelectrochemical Grätzel cells [15–16]. We argue here that efficient photocatalytic degradation of pollutants under visible light irradiation has to meet similar requirements to the ones of the dyes in Grätzel cells. In particular, the anchoring mode of the pollutant to the TiO_2_ surface influences the electron transfer [17]. The most commonly used anchoring group is the carboxylic acid group (–COOH) [16]. It ensures strong binding of the dye on the surface and promotes the charge transfer. The anchoring of the salicylate group on TiO_2_ has also been studied [18–19]. At the surface, both substituent groups of a benzene derivative are involved in the complexation of colloidal titanium dioxide [18]. This results in the formation of a six-atom ring with a chelating type of bonding to the same Ti(IV) ion. Similarly, the binding of the salicylic acid to titania was thought as bidentate chelate through the oxygen atoms of –OH and of –OCOH [14,20].

Theoretically, density functional theory (DFT) calculations showed [21–23] that the binding of the carboxy group to titania is bidentate bridging, with the monodentate anchoring being less stable [24–27]. The higher performance of the dyes with both carboxy and hydroxy anchoring groups [28] has led us to revisit earlier studies of a dye with three types of anchoring groups: –OH, –COOH and –SO_3_H [29–30]. We showed that although the salicylate does use both the carboxy and hydroxy substituent groups, the binding configuration is not bidentate chelate, as previously thought [14,18–20].

Building upon the experience gained while modeling materials for photoelectrochemical cells, we report here results of DFT and time dependent DFT (TD-DFT) calculations performed on several colorless aromatic pollutants, as well as complex systems consisting of benzene derivatives adsorbed on a TiO_2_ nanocluster. To answer the questions raised above we determine the electronic structure and the optical spectra of the pollutant itself, and find where the deprotonation is more likely to take place. We also simulate the pollutant–catalyst system to analyze the binding configurations. We discuss the energy level alignment between the pollutant and the catalyst as well as the charge transfer between the pollutants and the oxide. We compare our theoretical results with the experimental data available, particularly with the work of Wang et al. [14] on phenol (Ph), benzoic acid (BA), *p*-hydroxybenzoic acid (pHBA) and salicylic acid (SA), which attempted a correlation between the efficiency of photocatalytic degradation and the chemical structure of the pollutants over TiO_2_.

## Results and Discussion

This section is divided in five parts. The first describes the computational details whereas the second focuses on the optimized geometry and electronic structure of the free pollutants. The third subsection presents the binding of the pollutants to the titania nanocluster, the fourth presents the optical properties of the adsorbed pollutants, and the last subsection attempts to explain the experimental data as well as the key requirements for efficient photodegradation based on theoretical arguments.

### Computational details

The structures of all pollutants were optimized in neutral as well as deprotonated forms, using DFT [31–33], with the B3LYP exchange–correlation functional [34–35] and the double-ζ DZVP basis set including polarization functions for the valence electrons [36] of the free pollutants. In the case of the more complex pollutant–catalyst system the less demanding 3-21G(d) basis set was used for geometry optimization. All optimized structures were checked for stability by means of vibrational analyses. Time-dependent DFT [37] calculations of the molecular orbitals and the electronic transitions were performed in water by means of the polarizable continuum model (PCM) [38–39]. We used the same B3LYP functional and TZVP basis sets [36]. In the case of the pollutants adsorbed on the catalyst, the electronic states were accurately computed by using DZVP basis sets [40]. The Gaussian03 package [41] was used in all calculations.

### Free pollutants – electronic structure and optical properties

**Figure 1:**
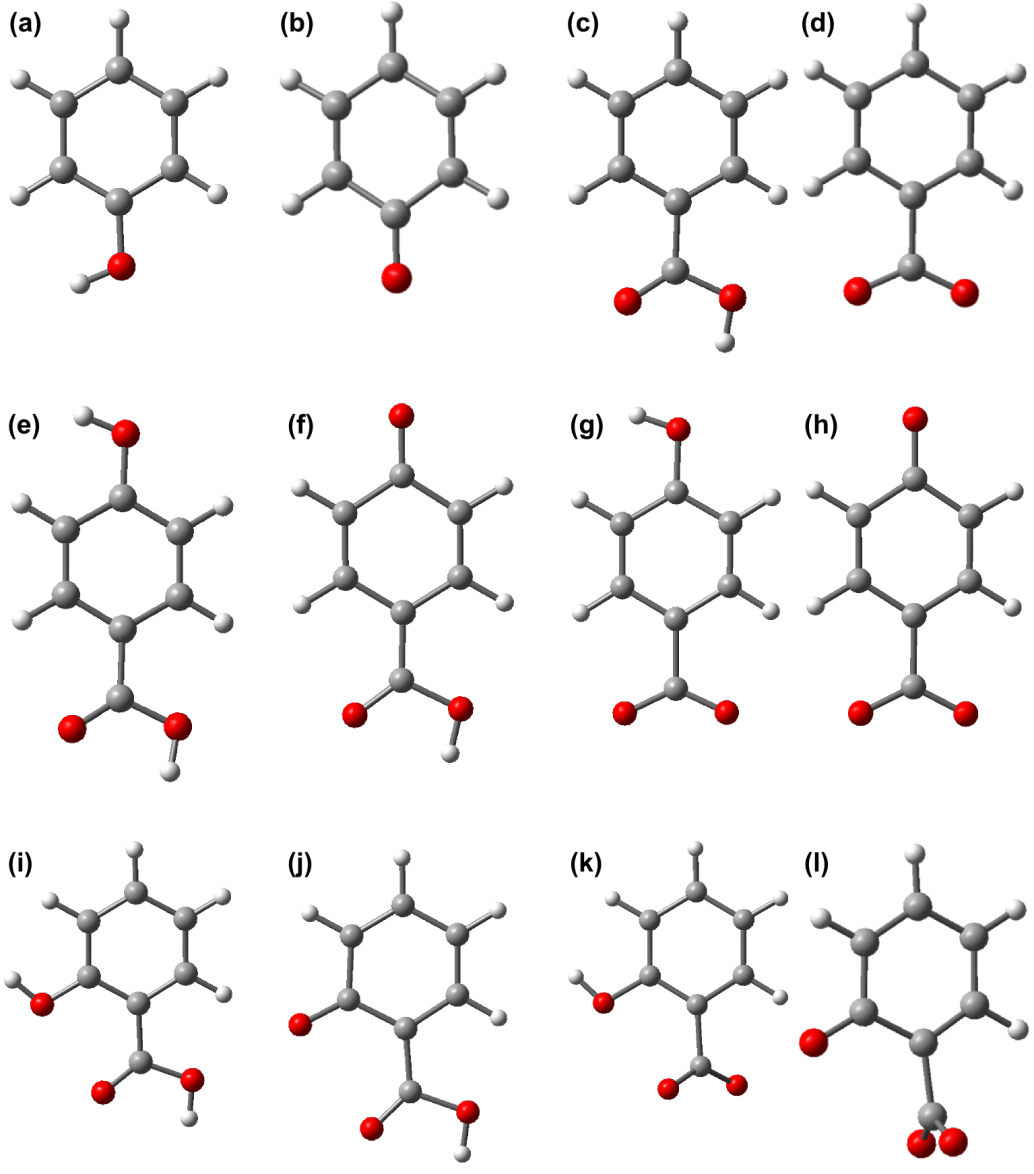
Optimized structure of pollutants in neutral and deprotonated forms calculated at DFT/B3LYP/DZVP level: a) neutral and b) deprotonated Ph, c) neutral and d) deprotonated BA, e) neutral, f) and g) deprotonated, h) doubly deprotonated pHBA, i) neutral, j) and k) deprotonated, l) doubly deprotonated SA.

During the photocatalytic degradation the benzene derivatives (phenol, Ph, benzoic acid, BA, *p*-hydroxybenzoic acid, pHBA, and salicylic acid, SA) undergo various processes, including deprotonation. We first perform a comparative analysis of the likelihood of the deprotonation process. For that, we found the optimized geometries and determined the total energy for all four aromatic pollutants in neutral and their deprotonated forms. The simulations of the deprotonated forms were performed by taking away a proton from the anchoring group, as it generally happens when the molecule is bound to the substrate. The optimized structures are shown in Figure 1.

Obvious differences between the geometries of the neutral and deprotonated forms are observed for salicylic acid. In the case of doubly deprotonated SA the carboxy group moves away from the plane of the aromatic ring by 86.2°, whereas for the form that is singly deprotonated at the carboxy group the torsion angle is 48.7°. A more careful analysis also reveals some slight differences between the bond lengths and some small distortions of the bond angles but these aspects are not of crucial importance in the following. The key question is at which anchoring group the deprotonation is more likely to take place. To find the answer we can look at the total energy of the pollutants as well as at the proton affinity, *PA*, equal to the difference between the energy of the deprotonated form and the energy of the neutral compound [42], reported in Table 1. For both pollutants, the lowest energy between the two singly deprotonated forms is obtained when the hydrogen atom of the carboxy group is removed.

**Table 1:** Proton affinities of pHBA and SA in neutral and various deprotonated forms, in water, based on DFT calculations at B3LYP/TZVP level. The labeling corresponds to the one used in Figure 1.

**pHBA**	(e)	(f)	(g)	(h)

*PA* (eV)	—	12.97	12.81	26.00

**SA**	(i)	(j)	(k)	(l)

*PA* (eV)	—	13.01	12.78	26.09

**Figure 2:**
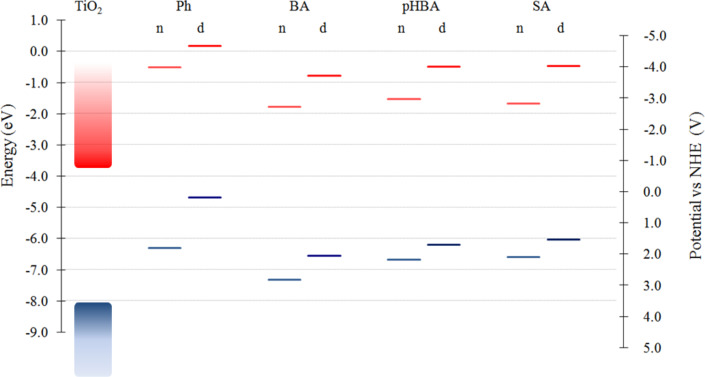
Diagram of energy with respect to vacuum as well as potential versus the normal hydrogen electrode (NHE) of pollutants in both neutral and deprotonated forms, calculated at DFT/B3LYP/TZVP level in solution. In the case of pHBA and SA the deprotonation is on the carboxy group.

The DFT calculated energies of the key molecular orbitals of the pollutants in neutral and deprotonated forms are represented in the diagram shown in Figure 2. To put everything in perspective we also represented the results of DFT calculations for TiO_2_ clusters [23], particularly the edges of the valence and conduction bands of titania. As discussed in [23,43], and the references therein, DFT calculations on finite size clusters tend to overestimate the gap with respect to the experimental values. It can be seen that the energy difference between the lowest unoccupied molecular orbital (LUMO) and the highest occupied molecular orbital (HOMO) is larger for all pollutants than the calculated band gap of the semiconductor. As a result, the absorption spectra of the free pollutants have peaks further in the UV region than the catalyst. Another important observation is related to the energy level alignment between the pollutants and the catalyst. As all LUMOs of the pollutants are above the conduction band edge of TiO_2_, the charge transfer to the semiconductor is possible. We note that the energies of the deprotonated forms are shifted upwards with respect to the neutral pollutants, as expected due to the missing proton.

**Figure 3:**
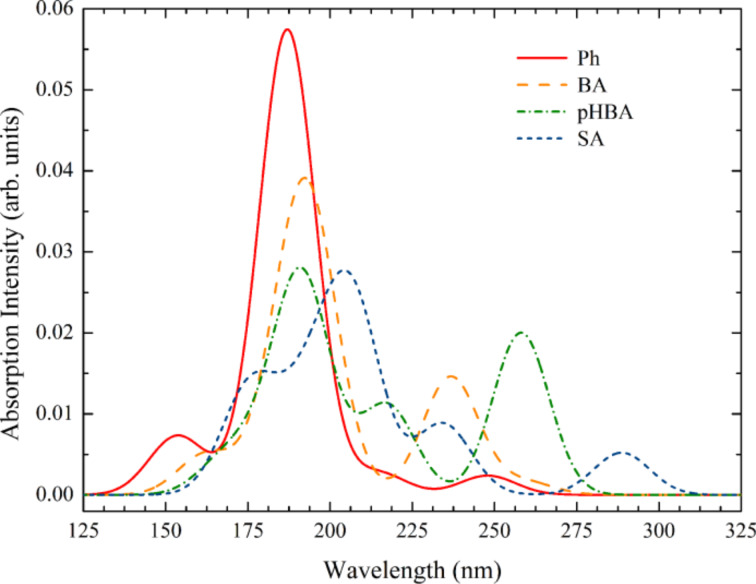
Simulated UV–vis absorption spectra of the first 10 transitions for neutral pollutants in water, calculated by TD-DFT. The spectral lines were convoluted with Gaussian distributions of 20 nm linewidth at half maximum.

The electronic spectra of the pollutants, simulated by TD-DFT calculations, are displayed in Figure 3. The spectra are all in the UV, as expected since the pollutants are all colorless, and in agreement with the results of DFT calculations presented in Figure 2. The energy of the electronic transitions, obtained by TD-DFT is a better indicator of the gap than the LUMO–HOMO energy difference determined by DFT [23].

Of all pollutants, salicylic acid has peaks at higher wavelengths, just below 300 nm. The spectrum of Ph has a HOMO→LUMO transition at 248 nm, a HOMO→LUMO+1 transition at 215 nm and HOMO−1→LUMO transitions at 187 nm. In the case of BA the transitions are at 259 nm (HOMO→LUMO), 237 nm (HOMO−1→LUMO), and 192 nm (HOMO→LUMO+1). Similarly, for pHBA we found transitions at 258 nm (HOMO→LUMO), 217 nm (HOMO→LUMO+1), and 191 nm (HOMO−1→LUMO+1). Finally, in the case of SA the spectrum is closest to the visible range: 289 nm (HOMO→LUMO), 234 nm (HOMO−1→LUMO), and 207 nm (HOMO→LUMO+1). All transitions have π–π* character, for all pollutants. The HOMO→LUMO transitions are weak, the most intense peaks being located for all four compounds in the range of 187–207 nm. These strong transitions involve levels just below HOMO and/or just above LUMO.

**Figure 4:**
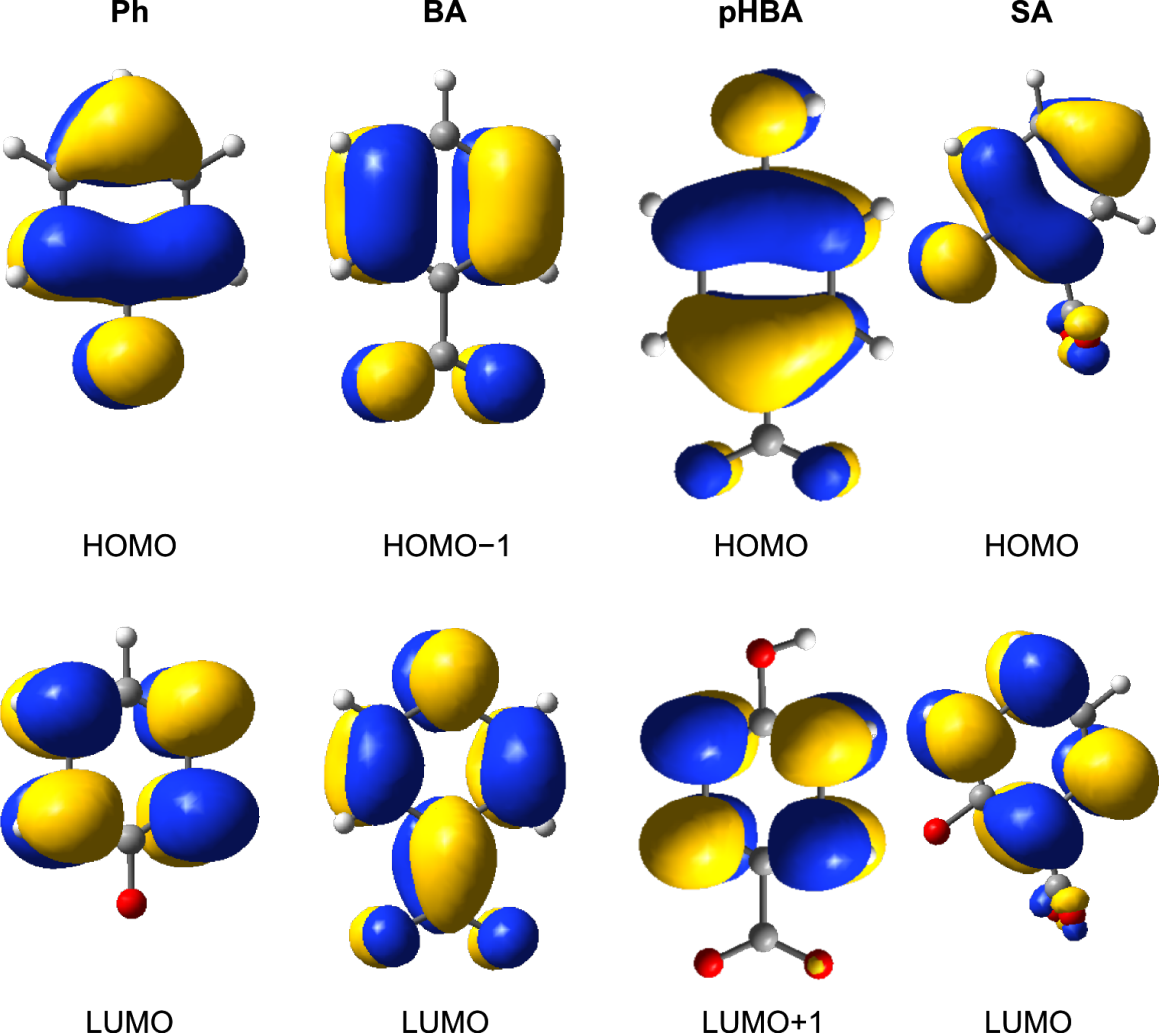
Isodensity surfaces (0.03 *e*/bohr^3^) of the key molecular orbitals of the deprotonated pollutants, calculated at DFT/B3LYP/ TZVP level in water. In the case of pHBA and SA the deprotonation is on the carboxy group.

Before ending this section we present in Figure 4 the key molecular orbitals of the pollutants, as they will be useful in identifying in a later section the MOs of the complex pollutant–catalyst system.

### Pollutant adsorption – binding configurations

As argued in the Introduction, the anchoring modes of the pollutants to the catalyst surface are of crucial importance for the charge transfer. But before discussing adsorption, a few comments regarding the titania cluster are in place. We model TiO_2_ nanoparticles by a geometry optimized cluster with the molecular formula Ti_24_O_50_H_4_. Prior to optimization, the cluster was cut from the experimental anatase structure with (101) and (001) surfaces [44]. The optimization led to some slight distortions from the lattice geometry, which lowered the surface energy. To prevent the occurrence of dangling bonds for some peripheric oxygen atoms and to avoid the problem of the surface states in the gap [45] we introduced four hydrogen atoms [29,46]. The resulting Ti_24_O_50_H_4_ cluster has a length of 12.76 Å and a width of 7.39 Å and provides a reasonable compromise between accuracy and computational costs given the small size of the pollutant molecules [29]. One last comment on this topic is related to the nature of the states in the valence and conduction band of titania. The valence band is dominated by the contributions from the p orbitals of oxygen, whereas the conduction band is dominated by the d orbitals of the Ti atoms [29,46].

**Figure 5:**
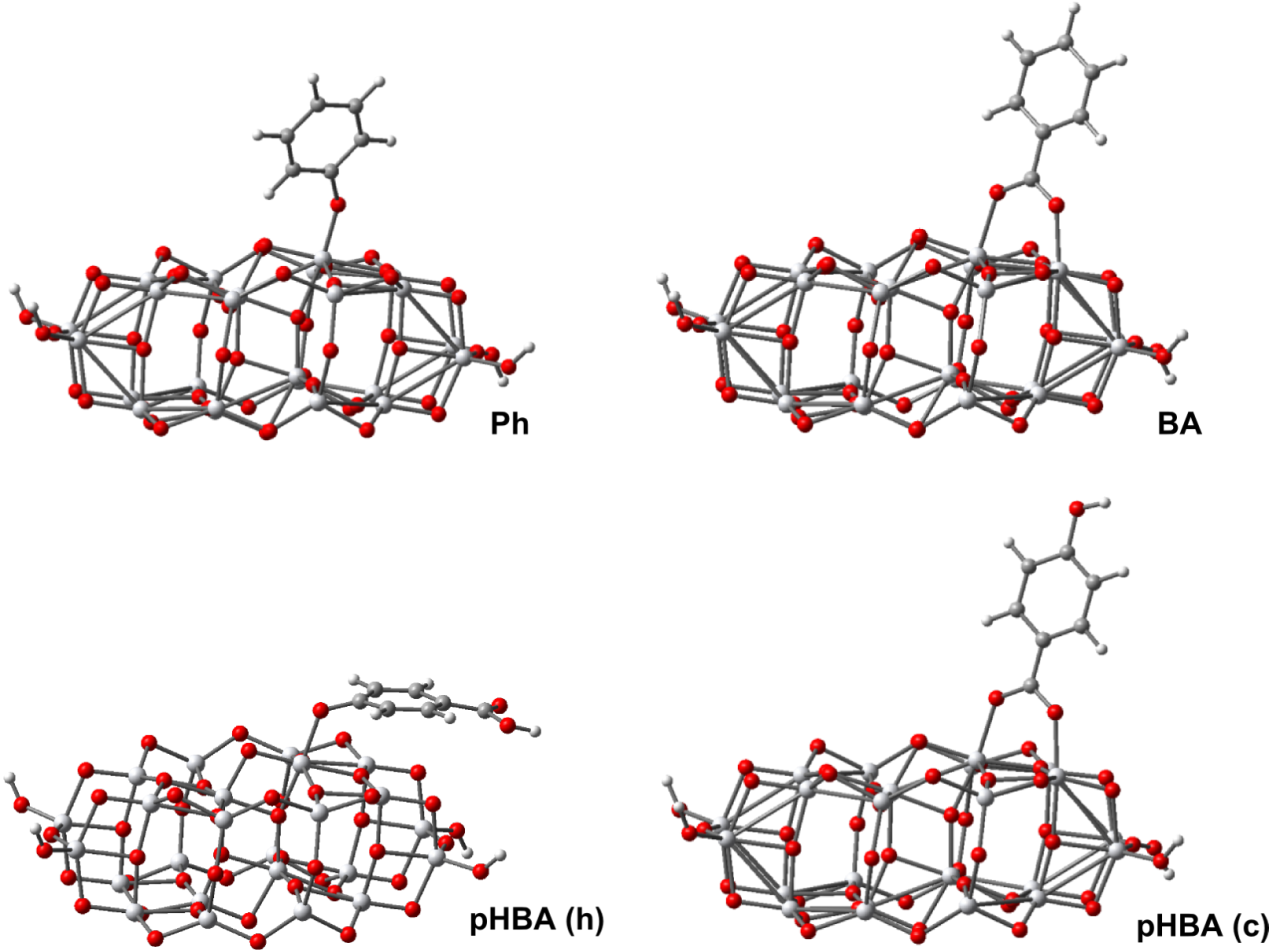
Optimized geometry of the pollutant-catalyst systems calculated at DFT/B3LYP/3-21G* level; pHBA is anchored either through the hydroxy (h) or the carboxy (c) groups.

We start our discussion of adsorption with Ph, which, after deprotonation, can bind to the (101) surface of the substrate through the oxygen atom. This is shown in Figure 5, which displays the optimized structure resulting from the DFT calculations. We note that the geometry optimization was performed for all pollutants starting with the molecule distanced from the cluster. The final geometry with pollutants bound to the Ti atoms of the (101) surface was robust with respect to variations in the initial configuration. The distances and angles relevant to the binding of Ph to the titania cluster are reported in Table 2. The dihedral angle τ, which is measured between the plane of the ring and the surface of the cluster (defined by three adjacent Ti ions), is 71.1° tilted away from the normal plane.

**Table 2:** Bond distances and bond angles relevant to the binding of the pollutants to the catalyst, after geometry optimization at DFT/B3LYP/3-21G* level; pHBA is anchored either through the hydroxy (h) or the carboxy (c) groups.

parameter	**Ph**	**BA**	**pHBA (h)**	**pHBA (c)**

*r*(Ti–O)	1.835 Å	—	1.940 Å	—
*r*(Ti–O1)	—	2.058 Å	—	2.049 Å
*r*(Ti–O2)	—	1.991 Å	—	1.985 Å
θ(Ti–O–C)	145.5°	—	120.2°	—
θ(Ti–O1–C)	—	128.3°	—	128.7°
θ(Ti–O2–C)	—	131.0°	—	131.0°
τ	71.1°	86.1°	13.6°	84.0°

In the case of BA, the adsorption is through the carboxy group. Experimental studies have shown that carboxylic acid groups can have various binding configurations with the Ti(IV) ions, ranging from monodentate ester-like binding [47], bidentate bridging [48], or both bidentate chelate and bridging [49–50]. However, theoretical calculations demonstrated that the preferred anchoring is bidentate bridging, with one proton transferred to a nearby surface oxygen [22,24–26]. Our calculations for BA are consistent with these earlier results, the bidentate bridging being the preferred adsorption mode, as shown in Figure 5b. The Ti–O bond distances reported in Table 2, are different, 2.058 Å and 1.991 Å the axis of the molecule being slightly tilted. The dihedral angle is 86.1° almost perpendicular to the surface of the catalyst.

For pHBA there are two possible anchors, one is the carboxy group, as in the case of BA, the other is the hydroxy group as for Ph. Our simulations provided different total energies for the two cases, the preferred binding being through the carboxy group, by an energy difference of about 1 eV. Such a result is not a surprise, as the strength of the mechanical adhesion is higher when two Ti–O bonds are involved instead of just one [45]. We also note the different orientation of the aromatic ring in the two cases. When the binding is through the carboxy group the aromatic ring is almost perpendicular to the surface of the catalyst (τ = 84.0°). In contrast, when the binding is of the (h)-type the ring is tilted almost parallel to the surface (τ = 13.6°), reminding of π stacking interactions.

**Figure 6:**
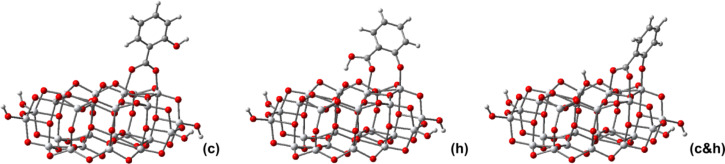
Optimized geometry of SA adsorbed on titania, calculated at DFT/B3LYP/3-21G* level.

In the case of SA adsorbed on TiO_2_ previous studies claimed that in the salicylate complex formed at the interface both substituent groups are involved, which leads to the formation of a six-atom ring with a chelating type of bonding to the same titanium ion [18–19]. Similarly, the binding of SA was thought as bidentate chelate through the oxygen atoms of –OH and of –COOH [14,20]. In a previous paper [29] we showed that for Mordant Yellow-10 anchoring through the SA part can lead to three binding modes, depending on the degree of deprotonation, but none of them was bidentate chelate. Returning to the present study, for SA we performed geometry optimization calculations in three cases. We deprotonated the carboxy group, case (c), the hydroxy group, case (h), and both, case (c&h), when we left one proton to find its equilibrium position on the TiO_2_ surface. All these cases are illustrated in Figure 6 and Table 3.

**Table 3:** Distances and angles relevant to the binding of SA to the catalyst, after geometry optimization at DFT/B3LYP/3-21G* level.

parameter	**SA (c)**	**SA (h)**	**SA (c&h)**

*r*(Ti–O)	—	1.850 Å	1.830 Å
*r*(Ti–O1)	2.051 Å	—	2.017 Å
*r*(Ti–O2)	1.969 Å	2.031 Å	2.036 Å
*r*(O–H)	—	1.112 Å/1.386 Å	—
θ(Ti–O–C)	—	146.0°	163.2°
θ(Ti–O1–C)	128.1°	123.6°	121.2°
θ(Ti–O2–C)	133.0^o^	—	125.0°
τ	84.8°	45.0°	72.0°
τ′	—	24.6°	62.0°

The first question to answer is which of the three types of anchoring is most stable. Our DFT calculations showed the most stable configuration is (c&h). At about 0.58 eV higher lies the SA deprotonated at the hydroxy group (h), followed at 1.18 eV by the configuration obtained by deprotonation of the carboxy group (c). Consequently, we confirm our previous calculation on Mordant Yellow 10, showing that the deprotonation of both groups lowers the energy the most, allowing for stable ‘mechanical’ structures with three pillars of unequal bond lengths to three different but adjacent Ti(IV) ions. The shortest bond length is the one to the oxygen of the hydroxy group. The triple binding is allowed by a rotation of τ′ *=* 62.0° of the –COO^−^ group with respect to the aromatic plane. On the other hand, the benzene ring encloses a dihedral angle of 72.0° with the plane of the surface.

Next, when the deprotonation takes place at the hydroxy group alone, a second bond is formed through the oxygen atom of the carboxy group and the third is a hydrogen bond involving the same carboxy group. We clearly demonstrate that the ‘common knowledge’ that the binding configuration is in a six ring chelate, suggested by infrared spectroscopy measurements [19–20] or simply assumed by other authors [14,18], is wrong. There are two direct bonds to two different Ti(IV) ions and one indirect bond to an oxygen through the H atom. Again, the third bond is allowed by a rotation of the –COO^−^ group by τ′ *=* 24.6°. Overall, the plane of the ring is tilted by 45.0° with respect to the surface of the catalyst. The last case involves only the usual bidentate bridging through the carboxy group. This anchoring configuration is overall similar to what we observed for BA and pHBA (c), with comparable bond lengths, bond angles and dihedral angles.

### Adsorbed pollutants – optical properties

**Figure 7:**
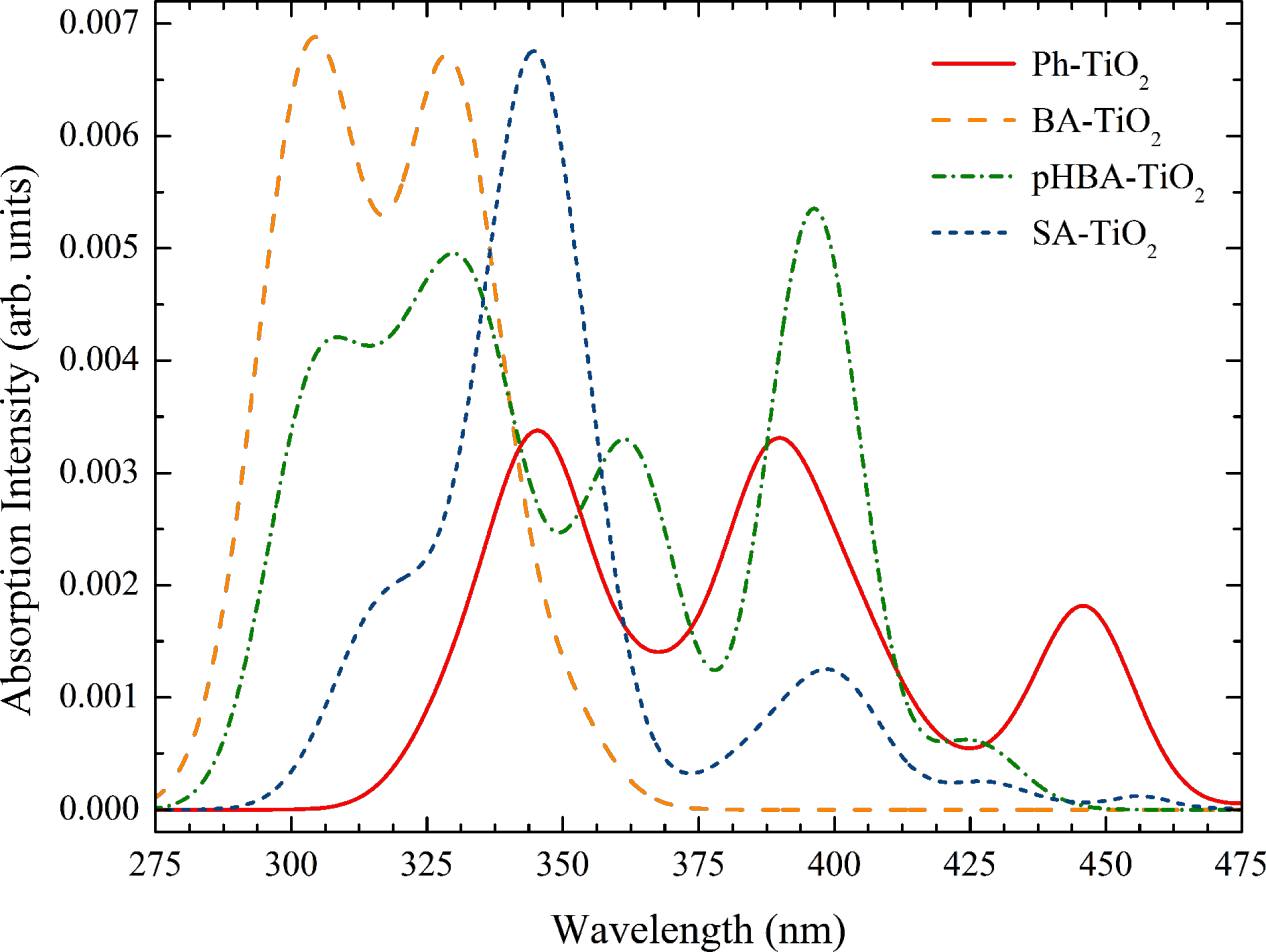
Simulated UV–vis absorption spectra of the pollutants bound to the TiO_2_ cluster, calculated by TD-DFT in water. The spectral lines were convoluted with Gaussian distributions of 20 nm linewidth at half maximum.

We recall our conclusion drawn based on Figure 2 and Figure 3, that the absorption bands of the free pollutants are situated further into the UV region than those of the catalyst. In contrast, the TD-DFT simulated optical spectra of the pollutants adsorbed onto the catalyst, displayed in Figure 7, show for all compounds a strong shift toward higher wavelengths. This red-shift is what makes possible the photocatalysis under visible light irradiation. In order to better understand why this red-shift takes place, it is useful to plot the densities of states for all adsorbed pollutants. But before that we take a more careful look at Figure 7.

The first (low energy/high wavelength) bands correspond for all systems to HOMO→LUMO transitions. However, these transitions have very small oscillator strengths, as it can be seen in Table 4. For instance, the only adsorbed pollutant with a band beyond the blue region of the spectrum is Ph, whose first transition is at 524 nm, but the intensity is extremely weak. The stronger UV–vis absorption bands of the adsorbed pollutants are located below 475 nm. Except for BA, whose key orbital seems to be HOMO-1, for the other adsorbed pollutants the transitions with noticeable oscillator strengths are from the HOMO to states above the conduction band edge, such as LUMO+2.

**Table 4:** Wavelength, oscillator strength and character of the most intense optical transition for the four pollutants adsorbed on the catalyst, calculated at TD-DFT/B3LYP/DZVP level in water.

pollutant	λ (nm)	*f*	character

**Ph**/Ti_24_O_50_H_4_	524	0.0002	HOMO→LUMO
	445	0.034	HOMO→LUMO+2
	387	0.038	HOMO→LUMO+13
	345	0.029	HOMO→LUMO+36

**BA**/Ti_24_O_50_H_4_	354	0.001	HOMO→LUMO
	348	0.019	HOMO−1→LUMO
	330	0.115	HOMO−1→LUMO+2
	306	0.041	HOMO−1→LUMO+4

**pHBA**/Ti_24_O_50_H_4_ (c)	425	0.013	HOMO→LUMO
	396	0.114	HOMO→LUMO+2
	365	0.040	HOMO→LUMO+6
	335	0.055	HOMO→LUMO+13
	303	0.018	HOMO→LUMO+29

**SA**/Ti_24_O_50_H_4_ (c&h)	454	0.002	HOMO→LUMO
	428	0.004	HOMO→LUMO+2
	399	0.014	HOMO→LUMO+8
	346	0.047	HOMO→LUMO+19
	319	0.008	HOMO−1→LUMO+6

For the adsorbed Ph we observe three major peaks, one in the visible range, at 445 nm, the other two in the UV range. In the case of the pHBA on the TiO_2_ nanocluster, the HOMO→LUMO band at 425 nm has a significant intensity, still much smaller than the next. At the limit of the visible range there is a transition at 396 nm, from HOMO to LUMO+2. When bound to the titania cluster, SA has two weak bands in the visible range and stronger absorption in the UV. Finally, the adsorbed BA has only absorption bands in the UV.

**Figure 8:**
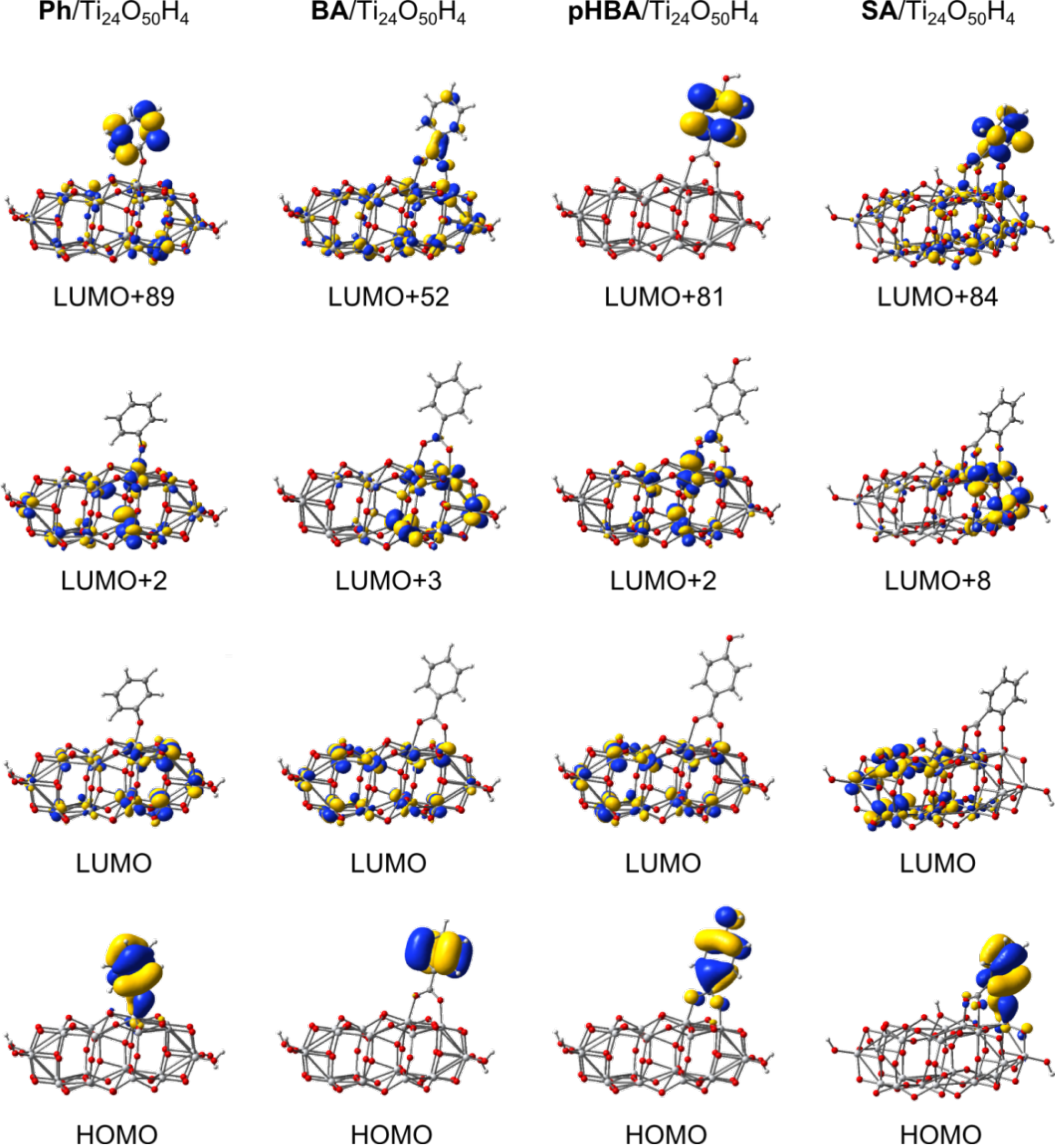
Isodensity surfaces (0.03 *e*/bohr^3^) of the key molecular orbitals of the deprotonated and adsorbed forms of pollutants, calculated at DFT/B3LYP/DZVP level in water.

In order to better understand the electronic spectra we looked at the electronic density of the key molecular orbitals, displayed in Figure 8. As expected, the HOMOs have most of the charge located on the pollutant, whereas the LUMOs correspond to the conduction band edge of the catalyst. The excited state of the adsorbed pollutant, identified by comparison with the electron densities of the free pollutants represented in Figure 4, is situated deep into the conduction band for all compounds. For instance, in the case of BA/Ti_24_O_50_H_4_ the corresponding MO is the 52nd from the LUMO, whereas in the other cases there are more than 80 states between the conduction band edge of titania and the excited state of the pollutant. Of crucial importance in the absorption spectra are the MOs mentioned in the transitions reported in Table 4. A common feature of these orbitals is the mixed character, the electron density extending from the semiconductor onto the pollutant, as it can be seen from the second row of Figure 8. In fact, it is this delocalization of the charge from the pollutant onto the catalyst in the case of the HOMOs as well as from the semiconductor to the adsorbed molecule, in the case of higher empty states, that make possible the higher wavelength electronic transitions and the absorption in the visible range. Although taken separately both the pollutants and the catalyst absorb in the UV, the states with mixed character lead to allowed optical transitions.

**Figure 9:**
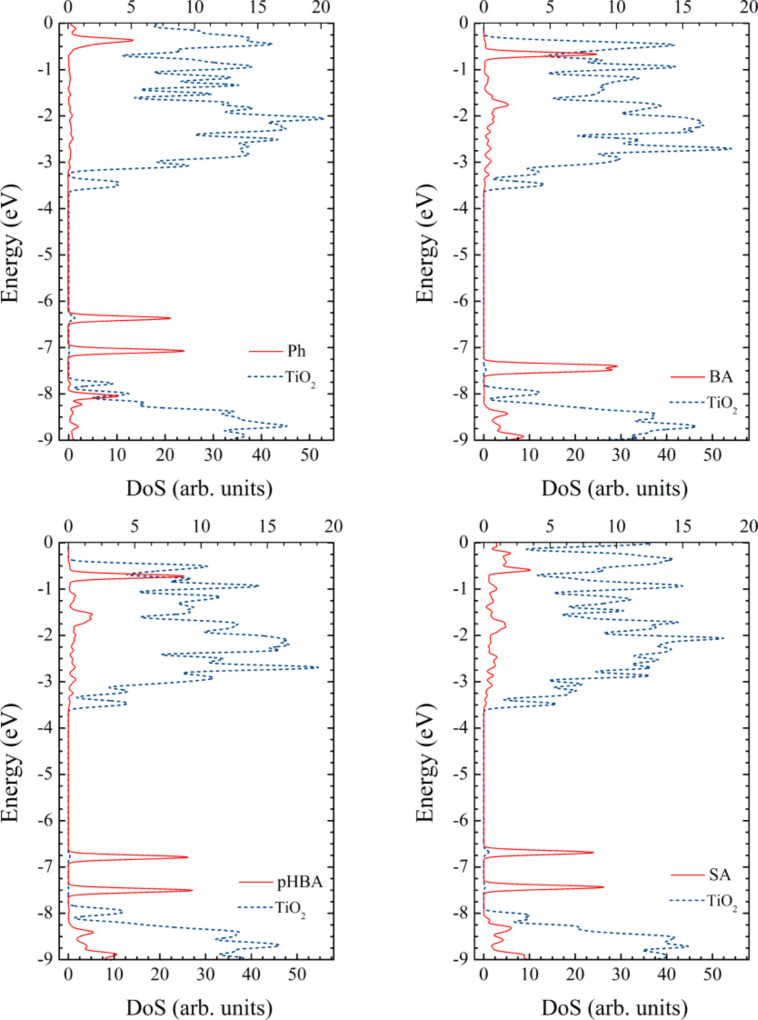
Density of states of the pollutant–catalyst complex systems, calculated at the DFT/B3LYP/DZVP level in water. Bottom scale is for the contribution of the Ti_24_O_50_H_4_ nanocluster (dotted line) whereas the top scale for the contribution of the pollutant (continues line). For all systems, the edge of the p-type valence band of the semiconductor is located at about −7.9 eV, whereas the d-type conduction band edge is situated at about −3.7 eV. The HOMOs of the pollutant are located in the gap of the semiconductor.

We can even better understand these concepts looking at the density of states (DOS) for the adsorbed pollutants presented in Figure 9. For all four systems, the densities of states have some common features. For instance, the edge of the p-type valence band is located just above −7.9 eV, whereas the d-type conduction band edge is situated just below −3.7 eV. We note that the DFT calculated gap of about 4.2 eV for TiO_2_ is overestimated with respect to the experimental value [23,43].

Common to all compounds is that in the gap, significantly higher than the valence band edge, there are two occupied states, HOMO−1 and HOMO, well localized on the pollutants. These two orbitals are separated by about 0.7 eV for three of the four systems, the only exception being BA, for which the two states are almost degenerate (the energy difference is only 0.08 eV). For the three systems for which the HOMO−1 is significantly below the HOMO, only the HOMO plays an important role in the optical spectra. In the fourth case, both HOMO−1 and HOMO can play a role but lying lower in energy, closer to the valence band edge, the energy difference is high and the transitions are in the UV. The energies of the HOMOs are: −6.36 eV, −7.39 eV, −6.78 eV and −6.68 eV for the complex systems with adsorbed Ph, BA, pHBA, and SA, respectively.

In line with the previous discussion, important to note is the mixed character of the states involved in the optical transitions. Looking carefully at the density of states, we can observe that the HOMOs have some small contribution from the catalyst and, similarly, the key states in the conduction band have a small contribution from the pollutant. The weak DOS peaks in the conduction band (note the different scale used) correspond to states with π* character and with sizeable electron density distributed over the pollutant, as shown in Figure 8.

For a more quantitative image we report in Table 5 the contributions to the electron density of the MOs involved in the optical transitions. This way we can see that the charge of the HOMOs is not entirely distributed on the pollutant. While BA keeps over 99% of the charge, the other pass more than 13%, 3%, and 11% (for Ph, pHBA, and SA, respectively) to titania. In the conduction band, the picture reverses, as most of the charge is on titania. However, even in this case there are states with a sizeable charge located on the pollutant.

**Table 5:** Contributions of pollutant, anchor group, and substrate, in %, to the electron density of MOs involved in lowest electronic transitions, calculated at DFT/B3LYP/DZVP level.

MO	pollutant	anchor	Ti_24_O_50_H_4_

**phenol**			

HOMO	86.95	19.09	13.05
LUMO	0.14	0.02	99.86
LUMO+2	1.29	0.63	98.71
LUMO+89	42.65	0.01	57.35

**benzoic acid**			

HOMO	99.58	0.67	0.42
LUMO	0.57	0.38	99.43
LUMO+2	0.15	0.01	99.85
LUMO+52	11.41	4.61	88.59

***p*-hydroxybenzoic acid**			

HOMO	96.29	8.86	3.71
LUMO	0.45	0.29	99.55
LUMO+2	0.04	0.001	99.96
LUMO+81	91.59	0.32	8.41

**salicylic acid**			

HOMO	88.33	21.84	11.67
LUMO	0.27	0.14	99.73
LUMO+8	2.48	1.49	97.52
LUMO+84	31.81	2.38	68.18

The data reported in Table 5 allow us to draw additional conclusions regarding the likelihood of the electron transfer. In Marcus’ theory of the electron transfer [51–53] an important factor in the expression of the electron transfer rate is the electronic matrix element describing the electronic coupling between the excited state of the pollutant and a state in the conduction band of the catalyst. When the orbitals of the two separate components are known, an indicator of the strength of the matrix element may be the overlap between those states. When the orbitals of the entire system are available, as it is the case in our work, we can alternatively look at the degree of mixing indicated by the electron density present on each component and at the electron density on the anchoring group that binds the pollutant to the surface of the catalyst. Taking another look at Figure 8, we note that the flow of charge from the pollutant to the catalyst has to take place through the anchor. If the electron density on the anchor is high in the donor state, the tendency for charge transfer has to be stronger. The states with little density on the atoms of the anchoring group (such as the HOMO of pHBA/TiO_2_, which has a nodal plane through the center of the carboxy group), are less likely to favor a charge transfer. Table 5 reports electron densities on the anchoring group for each pollutant–catalyst system. Comparing the MOs involved in the main optical transitions, LUMO+2 for Ph and LUMO+8 for SA, we see that the percentage of the charge localized on the pollutant and on the anchoring group is about two times larger for SA, suggesting a more efficient charge transfer.

One last comment in this section refers to the role of the vibronic coupling effects on the charge transfer to the TiO_2_ cluster. In the case of Grätzel cells it has been shown [54–55] that the kinetics of the electron transfer from an excited dye to the titania nanoparticle may be influenced by the vibrational motion of nuclei. The vibronic perturbation, due to the interplay of electron–electron interactions and the internal vibrations of the benzene derivative, may facilitate the charge transfer also in the case of photocatalytic degradation of the pollutants studied here.

### Discussion and comparison with experimental data

In this section we compare our theoretical results with the experimental data available, particularly with the work of Wang et al. [14], who showed that BA can hardly be degraded and, for the other three, the order of the degradation efficiency is: SA > Ph > pHBA. The questions we attempt to answer are: Why do some pollutants degrade faster than others? What are the requirements for an efficient photocatalytic degradation of the pollutants under visible light irradiation? We base our answer on an analogy with the photoelectrochemical Grätzel cells [15–16]. An efficient photocatalytic degradation under exposure to visible light requires: i) strong adsorption of the pollutant to the catalyst, ii) intense absorption of the pollutant or the combined pollutant–catalyst system in the visible range of the spectrum, iii) proper energy level alignment of the excited state of the pollutant and the conduction band edge of the catalyst, and iv) fast charge transfer from the pollutant to the catalyst. Other requirements would regard the chemical reactions that take place after the charge transfer but we will not address those issues here.

Starting with the first criterion, all four pollutants studied here can bind to the catalyst. However, the strength of the bond is not the same, as at one limit SA can exhibit a triple bond, whereas Ph can form only a single bond. Binding through the carboxy group is most likely bidentate bridging, in agreement with various theoretical observations [22,24–26,29,46] and in contrast to some other opinions [56]. Also, the binding of SA involves both substituent groups, but does not lead to the formation of a six-atom ring with a chelating type of bonding to the same Ti(IV) ion, as previously considered [14,18–20,57]. Instead, the proton migrates on the surface leaving all three oxygen atoms available for bonds to three adjacent Ti ions. Moreover, even if the proton were kept by the carboxy group, the binding would still not be chelate and an additional H-bond would be formed.

Checking the four colorless pollutants studied here against the second criterion, we note that BA/TiO_2_ has extremely poor absorption in the visible range. The other systems have some absorption bands at low wavelengths, due to the mixed character of the key MOs. For instance the HOMOs, localized mostly on the pollutant, have some small contribution from the catalyst, whereas for some states in the conduction band the situation is reversed. As a consequence, low energy transitions that were forbidden become allowed. Based strictly on the absorption (see Figure 7) we would be led to the conclusion that adsorbed Ph should degrade faster than SA (which has weak bands at 454 nm and 428 nm) and pHBA (425 nm).

The third criterion, asking that the excited state of the pollutant lies higher than the conduction band edge of the catalyst, is met by all four pollutants. Here, an observation regarding a running controversy may be useful, especially as it paves the way for the discussion of the next criterion. Using arguments based on Marcus’ theory of electron transfer and making some simplifying assumptions, it was suggested [58] that the larger the ‘driving force’ (i.e., the difference in energy between the excited state of the molecule and the conduction band edge of the semiconductor) the higher the injection rate. However, there are some experimental observations [29,59–62] backed by some arguments derived through molecular modeling [29,46], which contradict this claim, suggesting that in practice a large driving force is not necessarily a guarantee for high electron transfer rates.

The forth criterion, requiring a fast charge transfer from the pollutant to the substrate, is more difficult to analyze. One of the factors that influence the transfer rate is the matrix element, which, in turn, can be correlated with the overlap integral between the two states. The emphasis on the orbital overlap was underlined long ago [63] suggesting that the π* orbitals of the carboxy group would promote rapid electron injection into the conduction band of TiO_2_, due to its d-symmetry, but not that of SnO_2_ or ZnO, which have predominantly s-character. Keeping in mind that for Grätzel cells the charge transfer is optimized in the case of a strong overlap between the dye and the semiconductor [18], which is favored by the presence of charge on the anchor, by analogy, in the case of the pollutant–catalyst system, the electron density on the binding group can provide some information regarding the likelihood of the charge flow. We found that such a comparison favors SA against Ph and both against pHBA.

**Figure 10:**
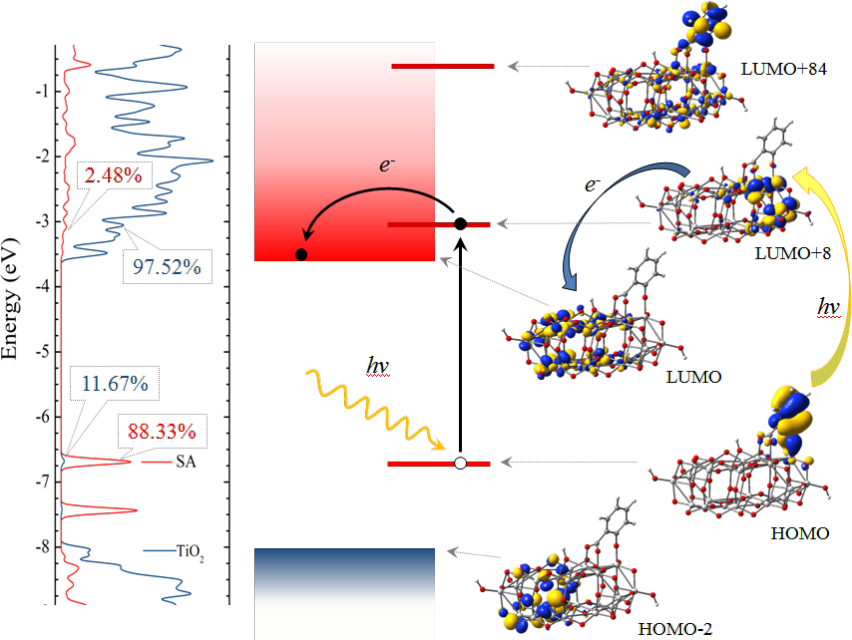
Schematic illustration of electro-optical processes that initiate the photocatalytic degradation of transparent pollutants on titania: (left) density of states, revealing the mixed character of the key MOs, (center) diagram showing the energy level alignment, the photoexcitation and the charge transfer, and (right) the molecular orbitals, showing the anchoring geometry and the charge localization.

A summary of the present discussion is displayed schematically in Figure 10. The left panel shows the density of states and reveals the mixed character of the key orbitals, such that, due to charge delocalization optical transitions in the visible become possible. The diagram in the central panel illustrates the energy level alignment and the position of the MOs involved in the excitation by absorption of visible light and in the electron transfer from the pollutant to the catalyst. Although typically the ‘driving force’ for the charge transfer is defined [53,58] as the difference between the energies of the excited state (LUMO+84, in our case) and of the conduction band edge (LUMO), here it may be more meaningful to consider the LUMO+8 orbital, which is much closer in energy and has a higher overlap with the LUMO. In the right panel the corresponding molecular orbitals are grouped to point out the electro-optical processes but they also presented the unusual anchoring, the charge delocalization as well as the pathways for charge flow.

Summing up, after checking whether the initial requirements for efficient photocatalytic degradation are met, we found that BA is disqualified by the poor absorption in the visible range. Of the other three, Ph has strongest optical absorption but it is outperformed by SA likely due to a more efficient electron transfer. We have to state that our approach cannot quantify the four different criteria discussed. Although the comparison with the experimental data is only qualitative, the present approach can explain some of the main features of the degradation curves observed.

## Conclusion

We reported results of DFT and TD-DFT calculations performed on several transparent aromatic pollutants as well as complex systems consisting of the benzene derivatives adsorbed on a TiO_2_ nanocluster. Our goal was to answer questions such as: Why can colorless pollutants degrade under visible light? Why do some pollutants degrade faster than others? To answer such questions we determined the electronic structure and the optical spectra of the pollutant itself and found where the deprotonation is more likely to take place. We optimized the geometry of pollutant–catalyst systems and shed some new light on the binding configurations of the benzene derivatives onto titania. We were able to dispel some misconceptions regarding the monodentate binding of the carboxy group, six-ring chelate binding of the salicylic acid. We demonstrated that the optimal binding configuration is bidentate bridging for the carboxy group and tridentate to adjacent titanium ions for SA.

We explained why transparent pollutants adsorbed onto a catalyst that absorbs only in the UV can degrade under visible light based on the mixed character of the key MOs involved in optical transitions. These orbitals are delocalized on both the pollutant and the catalyst such that some low energy transitions that were forbidden become allowed. We attempted to explain the experimental facts which state that the efficiency of degradation under visible light irradiation decreases in the sequence SA > Ph > pHBA > BA. Thus, analyzing the pollutants against some requirements for efficient photocatalytic degradation, we found that BA has no absorption in the visible range even bound to the catalyst. Ph has best light absorption but weakest anchoring, pHBA has very weak electron density on the anchor, which hinders the charge transfer, and that SA has strongest binding and offers the best pathways for charge flow.

## References

[1] Fujishima A, Zhang X, Tryk D A (2008). Surf Sci Rep.

[2] Frank S N, Bard A J (1977). J Am Chem Soc.

[3] Hoffmann M R, Martin S T, Choi W, Bahnemann D W (1995). Chem Rev.

[4] Linsebigler A L, Lu G, Yates J T (1995). Chem Rev.

[5] Friedmann D, Mendive C, Bahnemann D (2010). Appl Catal, B: Environ.

[6] Kumar S G, Devi L G (2011). J Phys Chem A.

[7] Liu G, Li X, Zhao J, Hidaka H, Serpone N (2000). Environ Sci Technol.

[8] Chen C, Li X, Ma W, Zhao J, Hidaka H, Serpone N (2002). J Phys Chem B.

[9] Agrios A G, Gray K A, Weitz E (2003). Langmuir.

[10] Agrios A G, Gray K A, Weitz E (2004). Langmuir.

[11] Kim S, Choi W (2005). J Phys Chem B.

[12] Li M, Tang P, Hong Z, Wang M (2008). Colloids Surf, A.

[13] Paul T, Miller P, Strathmann T J (2007). Environ Sci Technol.

[14] Wang N, Zhu L, Huang Y, She Y, Yu Y, Tang H (2009). J Catal.

[15] Grätzel M (2001). Nature.

[16] Hagfeldt A, Boschloo G, Sun L, Kloo L, Pettersson H (2010). Chem Rev.

[17] Lungu J, Oprea C I, Dumbrava A, Enache I, Georgescu A, Rădulescu C, Ioniţă I, Cimpoca G V, Gîrţu M A (2010). J Optoelectron Adv Mater.

[18] Moser J, Punchihewa S, Infelta P P, Gratzel M (1991). Langmuir.

[19] Tunesi S, Anderson M A (1992). Langmuir.

[20] Tunesi S, Anderson M (1991). J Phys Chem.

[21] Vittadini A, Selloni A, Rotzinger F P, Grätzel M (2000). J Phys Chem B.

[22] Pastore M, De Angelis F (2012). Phys Chem Chem Phys.

[23] De Angelis F, Fantacci S, Selloni A (2008). Nanotechnology.

[24] Srinivas K, Yesudas K, Bhanuprakash K, Rao V J, Giribabu L (2009). J Phys Chem C.

[25] León C P, Kador L, Peng B, Thelakkat M (2006). J Phys Chem B.

[26] Martsinovich N, Troisi A (2011). J Phys Chem C.

[27] Hagberg D P, Yum J H, Lee H J, De Angelis F, Marinado T, Martin Karlsson K, Humphry-Baker R, Sun L, Hagfeldt A, Grätzel M (2008). J Am Chem Soc.

[28] Chen Y-S, Li C, Zeng Z-H, Wang W-B, Wang X-S, Zhang B-W (2005). J Mater Chem.

[29] Oprea C I, Dumbravă A, Enache I, Lungu J, Georgescu A, Moscalu F, Oprea C, Gîrţu M A (2011). Phys Status Solidi A.

[30] Oprea C I, Panait P, Lungu J, Stamate D, Dumbravă A, Cimpoesu F, Gîrţu M A (2013). Int J Photoenergy.

[31] Hohenberg P, Kohn W (1964). Phys Rev.

[32] Kohn W, Sham L J (1965). Phys Rev.

[33] Parr R G, Yang W (1989). Density-Functional Theory of Atoms and Molecules.

[34] Becke A D (1993). J Chem Phys.

[35] Lee C, Yang W, Parr R G (1988). Phys Rev B.

[36] Rassolov V A, Ratner M A, Pople J A, Redfern P C, Curtiss L A (2001). J Comput Chem.

[37] Casida M E, Jamorski C, Casida K C, Salahub D R (1998). J Chem Phys.

[38] Barone V, Cossi M (1998). J Phys Chem A.

[39] Tomasi J, Mennucci B, Cammi R (2005). Chem Rev.

[40] Godbout N, Salahub D R, Andzelm J, Wimmer E (1992). Can J Chem.

[41] (2004). Gaussian 03.

[42] Wang Z, Chu I K, Rodriquez C F, Hopkinson A C, Michael Siu K W (1999). J Phys Chem A.

[43] Lundqvist M J, Nilsing M, Persson P, Lunell S (2006). Int J Quantum Chem.

[44] Selloni A (2008). Nat Mater.

[45] Wahab H S, Bredow T, Aliwi S M (2008). Chem Phys.

[46] Oprea C I, Panait P, Cimpoesu F, Ferbinteanu M, Gîrţu M A (2013). Materials.

[47] Falaras P (1998). Sol Energy Mater Sol Cells.

[48] Duffy N W, Dobson K D, Gordon K C, Robinson B H, McQuillan A J (1997). Chem Phys Lett.

[49] Ma T, Inoue K, Yao K, Noma H, Shuji T, Abe E, Yu J, Wang X, Zhang B (2002). J Electroanal Chem.

[50] Weng Y-X, Li L, Liu Y, Wang L, Yang G-Z (2003). J Phys Chem B.

[51] Marcus R A, Sutin N (1985). Biochim Biophys Acta.

[52] Gao Y Q, Georgievskii Y, Marcus R A (2000). J Chem Phys.

[53] Pelet S, Moser J-E, Grätzel M (2000). J Phys Chem B.

[54] Minaev B F, Minaeva V A, Baryshnikov G V, Girtu M A, Agren H (2009). Russ J Appl Chem.

[55] Baryshnikov G V, Minaev B F, Minaeva V A (2011). Opt Spectrosc.

[56] Tachikawa T, Yoshida A, Tojo S, Sugimoto A, Fujitsuka M, Majima T (2004). Chem–Eur J.

[57] Tachikawa T, Tojo S, Fujitsuka M, Majima T (2004). Chem Phys Lett.

[58] Anderson N A, Lian T (2004). Coord Chem Rev.

[59] Hara K, Sato T, Katoh R, Furube A, Ohga Y, Shinpo A, Suga S, Sayama K, Sugihara H, Arakawa H (2003). J Phys Chem B.

[60] Zhang X, Zhang J-J, Xia Y-Y (2008). J Photochem Photobiol, A: Chem.

[61] Sánchez-de-Armas R, San Miguel M Á, Oviedo J, Sanz J F (2012). Phys Chem Chem Phys.

[62] Chen R, Yang X, Tian H, Wang X, Hagfeldt A, Sun L (2007). Chem Mater.

[63] Anderson S, Constable E C, Dare-Edwards M P, Goodenough J B, Hamnett A, Seddon K R, Wright R D (1979). Nature.

